# Single-cell transcriptomics reveals gene signatures and alterations associated with aging in distinct neural stem/progenitor cell subpopulations

**DOI:** 10.1007/s13238-017-0450-2

**Published:** 2017-07-26

**Authors:** Zhanping Shi, Yanan Geng, Jiping Liu, Huina Zhang, Liqiang Zhou, Quan Lin, Juehua Yu, Kunshan Zhang, Jie Liu, Xinpei Gao, Chunxue Zhang, Yinan Yao, Chong Zhang, Yi E. Sun

**Affiliations:** 1 Stem Cell Translational Research Center, Tongji Hospital, Tongji University School of Medicine, Shanghai, 200065 China; 2 Department of Psychiatry and Biobehavioral Sciences and Intellectual Development and Disabilities Research Center, David Geffen School of Medicine, University of California, Los Angeles, CA 90095 USA; 3 Collaborative Innovation Center for Brain Science, Tongji University, Shanghai, 200092 China

**Keywords:** NSC/NPCs, SEZ/SVZ, single cell transcriptome, aging, cell cycle, Erk1/2

## Abstract

Aging associated cognitive decline has been linked to dampened neural stem/progenitor cells (NSC/NPCs) activities manifested by decreased proliferation, reduced propensity to produce neurons, and increased differentiation into astrocytes. While gene transcription changes objectively reveal molecular alterations of cells undergoing various biological processes, the search for molecular mechanisms underlying aging of NSC/NPCs has been confronted by the enormous heterogeneity in cellular compositions of the brain and the complex cellular microenvironment where NSC/NPCs reside. Moreover, brain NSC/NPCs themselves are not a homogenous population, making it even more difficult to uncover NSC/NPC sub-type specific aging mechanisms. Here, using both population-based and single cell transcriptome analyses of young and aged mouse forebrain ependymal and subependymal regions and comprehensive “big-data” processing, we report that NSC/NPCs reside in a rather inflammatory environment in aged brain, which likely contributes to the differentiation bias towards astrocytes versus neurons. Moreover, single cell transcriptome analyses revealed that different aged NSC/NPC subpopulations, while all have reduced cell proliferation, use different gene transcription programs to regulate age-dependent decline in cell cycle. Interestingly, changes in cell proliferation capacity are not influenced by inflammatory cytokines, but likely result from cell intrinsic mechanisms. The Erk/Mapk pathway appears to be critically involved in regulating age-dependent changes in the capacity for NSC/NPCs to undergo clonal expansion. Together this study is the first example of using population and single cell based transcriptome analyses to unveil the molecular interplay between different NSC/NPCs and their microenvironment in the context of the aging brain.

**Electronic supplementary material:**

The online version of this article (doi:10.1007/s13238-017-0450-2) contains supplementary material, which is available to authorized users.

## Introduction

Adult neural stem/progenitor cells (NSC/NPCs) exist in the subependyma/subventricular zone (SEZ/SVZ) of the forebrain as well as the subgranular zone (SGZ) of hippocampus (Zhao et al., 2008). These NSC/NPCs support the on-going adult neurogenesis throughout life to replenish olfactory bulb inter-neurons as well as granule neurons in hippocampal dentate gyrus (Ming and Song, 2011). Recently, our single cell transcriptome analyses revealed that CD133 positive ependymal cells throughout the whole ventricular surface of the central nervous system (CNS) harbored quiescent neural stem cell properties, which upon VEGF stimulation can be mitotically activated, and with subsequent bFGF treatment, enter a transit amplifying stage and then differentiate into neurons and glia in CNS regions that were not known to harbor adult neurogenesis (Luo et al., 2015). Since VEGF is abundant after CNS injury, we propose that CD133 positive ependymal cells are mitotically activated after injury, and with proper cellular environment, these cells are capable for neural repair (Yang et al., 2015; Duan et al., 2015).

Adult NSC/NPCs reside in highly complex cellular environment and themselves are also highly heterogeneous cell populations (Beckervordersandforth et al., 2010; Coskun et al., 2008; Morrison and Spradling, 2008). When it comes to investigating alterations in molecular characteristics associated with aging in distinct subpopulations of adult NSC/NPCs, conventional RNA sequencing becomes insufficient, because it only reflects the sum and/or average molecular features of all cells (Shapiro et al., 2013; Nolan et al., 2013). To resolve such a problem, single-cell-based transcriptome analyses are essential. Single-cell RNA-seq can precisely reflect the molecular characteristics of individual cells, which might be hidden from total-cell RNA-seq, if these features belong to rare cells in heterogeneous populations (Shalek et al., 2013). However, these features may be of particular importance for critical biological functions. Because of the high resolution of single cell transcriptome analyses, new subtypes of adult NSC/NPCs were gradually discovered in mouse SEZ/SVZ (Llorens-Bobadilla et al., 2015; Dulken et al., 2017). Single-cell transcriptome analysis, being able to reveal the molecular heterogeneity among cells, has been employed to demonstrate dynamic alterations in various biological processes and innate signals (Luo et al., 2015; Kim DH et al., 2015).

Aging related cognitive decline has recently been attributed, at least in part, to reduced NSC/NPC activities. With progression of aging, adult neurogenesis gradually declines and the number of NSC/NPCs continues to decrease in mouse SEZ/SVZ (Enwere et al., 2004; Maslov et al., 2004). Our previous work and others’ have shown that there are at least three quiescent and three active subpopulations of NSCs in mouse ependymal zone (EZ) or SEZ/SVZ (Llorens-Bobadilla et al., 2015; Luo et al., 2015; Dulken et al., 2017). Whether similar or different molecular mechanisms underlying distinct NSC/NPC subtypes during aging remains unclear.

In current study, by both population-based and single-cell transcriptome analysis, we identified three subpopulations of NSC/NPCs that expressed distinct combination of NSC/NPC markers in young and aged mouse SEZ/SVZ. It became clear that a handful of cell type specific markers were insufficient to define a homogenous cell population. Instead, the whole transcriptome or a large cluster of genes or a gene module, may be utilized to better define a distinct cell population. Moreover, we report here age-dependent transcriptional alterations in distinct NSC/NPC subpopulations as well as features of the microenvironment where these cells reside. Both cell extrinsic and intrinsic mechanisms underlying NSC/NPC aging were uncovered.

## Results

### NSC/NPCs from aged mice showed reduced proliferation capacity and neurogenic potentials

**Figure 1:**
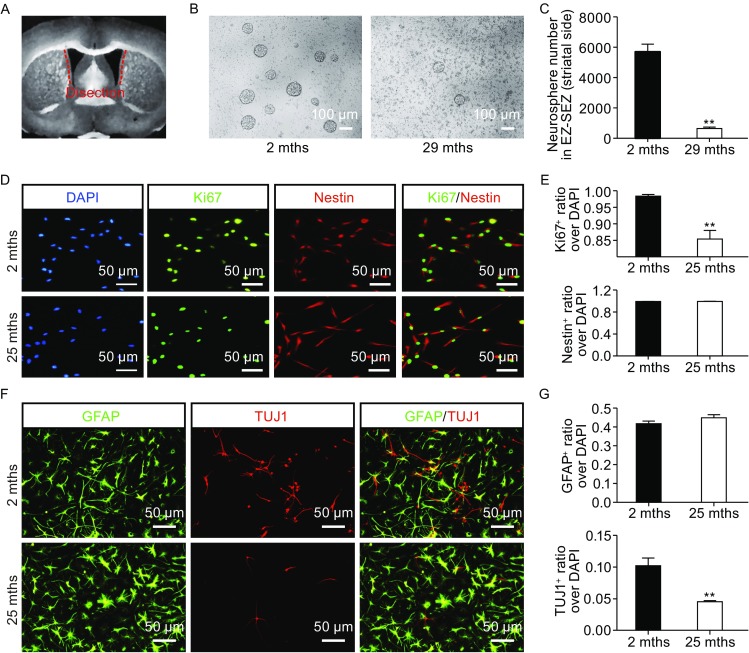
**Decreased proliferation and neurogenic potential of cultured NSC/NPCs derived from aged mouse SEZ/SVZ**. (A) Schematic showing SVZ dissection site. (B) Neurospheres clonally derived from NSC/NPCs from young (2 mths) and old (29 mths) mouse SEZ/SVZs. (C) Quantification of neurospheres in (B) (*n* = 3). (D) Immunofluorescence staining of Ki67 and Nestin in cultured NSC/NPCs from young (2 mths) and old (25 mths) mouse SEZ/SVZ. (E) Quantification of (D). (F) Representative fields of GFAP and Tuj1 immunofluorescence staining of cultured NSC/NPCs from young (2 mths) and old (25 mths) mice, after 7 days of spontaneous differentiation. (G) Quantification of (F). (**P < 0.01, *n* ≥ 3)

To determine the potential proliferation and differentiation capacities of aged NSC/NPCs in the SEZ/SVZ, we micro-dissected walls (EZ together with SEZ/SVZ) of lateral ventricles covering striatum and dissociated them into single cells (Fig. 1A). When plated on uncoated surfaces at a clonal density in the presence of fibroblast growth factor (bFGF), NSCs isolated from aged mouse (29 mths) SEZ/SVZ formed significantly less and smaller neurospheres than that from young mice (2 mths) (Fig. 1B and 1C). The number of Ki67^+^ proliferating cells was dramatically decreased in NSC/NPCs isolated and cultured from aged SEZ/SVZ (Fig. 1D and 1E), suggesting reduced cell cycling in aged NSC/NPCs. Upon differentiation, aged NSC/NPCs differentiated into less neurons, indicative of reduced neurogenic potentials (Fig. 1F and 1G).

**Figure 2:**
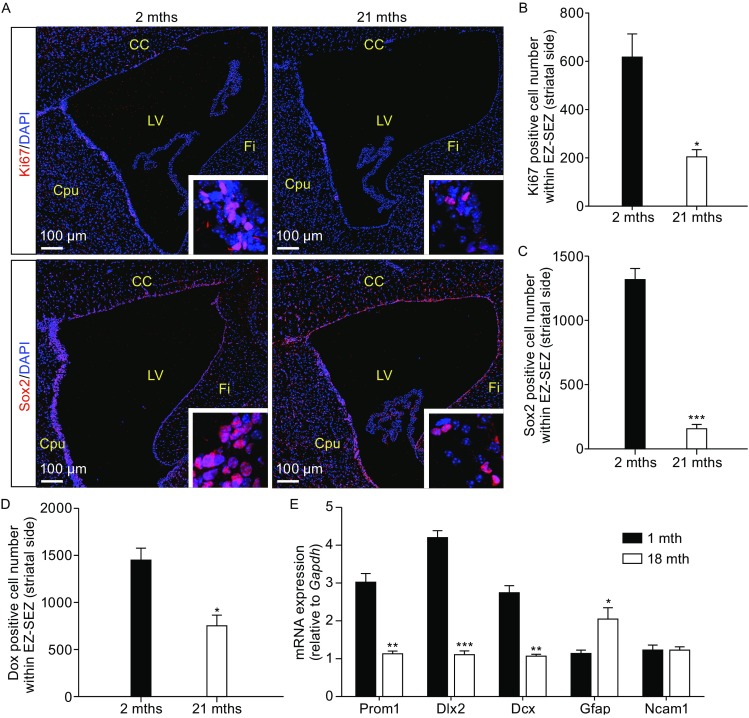
**Reduced cell proliferation and neurogenesis in aged mouse forebrain neurogenic zone**. (A) Micrographs of sagittal sections from young (2 mths) and old (21 mths) mouse EZ-SEZ/SVZ region immunostained with a proliferation marker Ki67 and a neural stem cell marker Sox2. The insets respectively indicate Ki67 and Sox2 positive cells. (B–D) Quantification of Ki67, Sox2, Dcx positive cells in young and old mouse EZ-SEZ/SVZ region lining the striatal side (*n* ≥ 3 per age group, see MATERIALS AND METHODS). (E) The mRNA expression of several NPC marker genes (*Prom1*, *Dlx2*, *Dcx*, *Gfap*, and *Ncam1*) were measured by qPCR of total RNA extracted from young (1 mth) and old (18 mths) mouse EZ-SEZ/SVZ tissues (*n* = 3 per group). Error bars indicate the SEM from three independent experiments. *P < 0.05; **P < 0.01; ***P < 0.001. CC, corpus callosum; Cpu, caudate putamen; LV, lateral ventricle; Fi, fimbria of the hippocampus

Consistent with *in vitro* NSC/NPCs culture results, numbers of Ki67 and Sox2 positive NSC/NPCs as well as doublecortin (Dcx)-positive neuroblasts, decreased in SEZ/SVZ of aged mice (Fig. 2A–D). Moreover, mRNA expression levels of an ependymal NSC marker, CD133, neuroblast markers, Dlx2 and Dcx, significantly decreased in SEZ/SVZ regions of aged mice (Fig. 2E), while Gfap, an astrocyte or NSC marker, increased expression at mRNA levels. Taken together, these observations suggest that aged SEZ/SVZ NSC/NPCs do decrease proliferation and neurogenic potential both *in vitro* and *in vivo*.

### The microenvironment of NSC/NPCs in aged brain is pro-inflammation

**Figure 3:**
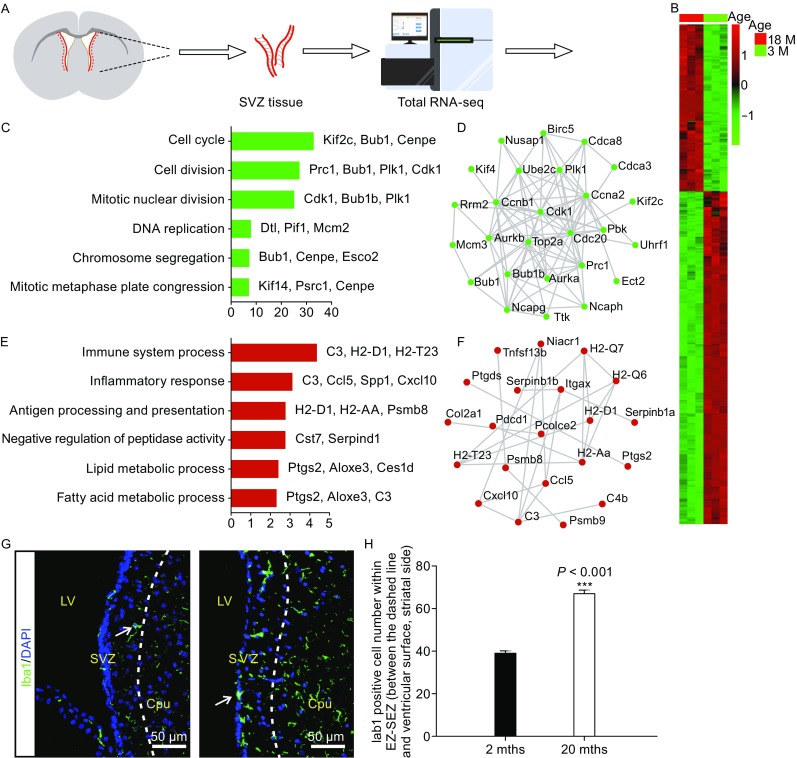
**Total RNA-seq of brain tissue surrounding EZ-SEZ/SVZ revealed NSC/NPCs reside in a rather inflammatory environment in aged brain**. (A) Schematic showing EZ-SEZ/SVZ tissue dissection and subsequent total RNA-seq. (B) Heatmap of differentially expressed genes in EZ-SEZ/SVZ tissue respectively derived from young (3 weeks) and old (18 mths) mice (*n* = 3 per age group). (C) GO analyses and (D) STRING protein-protein interaction network of down-related genes in aged group. Length of bars indicated the significance (−log10 transferred *P*-value, Fisher exact test). (E) GO analyses and (F) STRING protein-protein interaction network of up-related genes in old group. Length of bars indicated the significance (−log10 transferred *P*-value, Fisher exact test). (G) Representative micrograph of sagittal sections from young (2 mths) and old (20 mths) mouse SEZ/SVZ region immune-stained with Iba1 (green). (H) Quantification of Iba1 positive cells in young (2 mths, *n* ≥ 3) and old (20 mths, *n* ≥ 3) mouse SEZ/SVZ region. Error bars indicate the SEM from at least three independent experiments. ***P < 0.001. Cpu, caudate putamen; LV, lateral ventricle

To explore the molecular mechanisms underlying NSC/NPCs aging, we first performed transcriptome analyses of brain tissues surrounding EZ-SEZ/SVZ lining lateral ventricles covering striatum from young and aged mice (Fig. 3A). Differential gene expression clearly demonstrated that comparing to young mice, cell cycle and cell proliferation-related genes were downregulated in aged brain, consistent with reduced Ki67 immuno-labeling (Figs. 2A and 3B–D). In contrast, old mice had many immune response, inflammation-related genes elevated expression (Fig. 3B, 3E, and 3F), suggesting that aged brains possess a quite inflammatory microenvironment surrounding the EZ-SEZ/SVZ where adult neural stem cells reside. To confirm the gene expression result, we performed immuno-histochemical analyses to measure the amount of microglia, which are residential immune cells in the brain. It is obvious that within brain regions near EZ-SEZ/SVZ many IBA1 positive microglial cells were present, indicative of elevated inflammation (Fig. 3G and 3H). Our previous studies have demonstrated that inflammatory cytokines IL-6, IL-1β, LIF, CNTF, potently induce astrocyte differentiation or reactive gliosis through activation of the JAK-STAT pathway, while at the same time inhibit neuronal differentiation (Bonni et al., 1997; Sun et al., 2001). We speculate that the highly inflammatory microenvironment in aged brain contributes to the reduced neurogenic potentials of aged NSC/NPCs.

### Single cell RNA-seq of a discrete population of cells isolated from SEZ/SVZ adjacent to striatum in young and old mice

**Figure 4:**
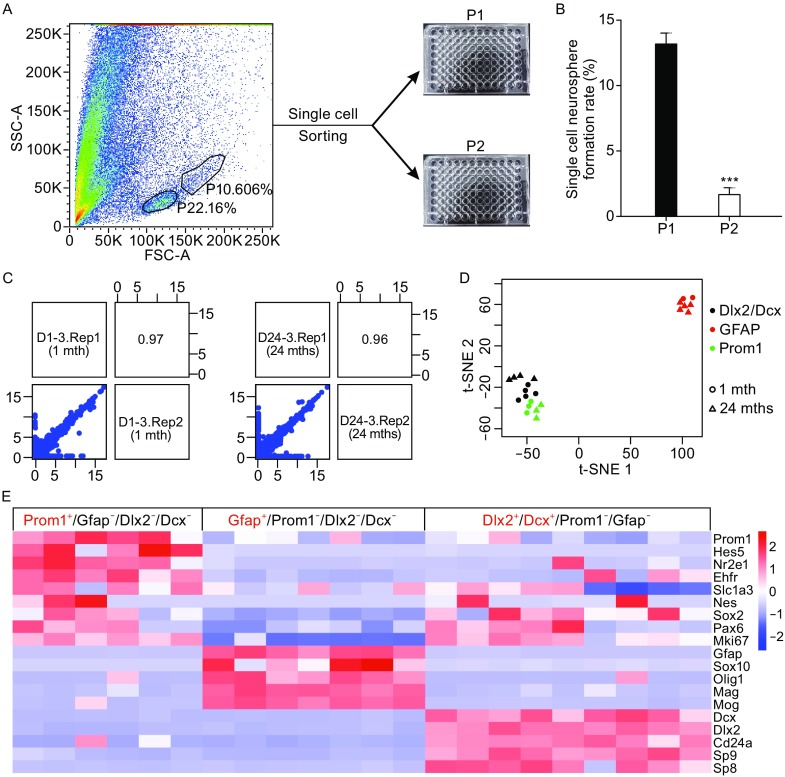
**Transcriptome analysis of single cells derived from a discrete SEZ/SVZ population of cells**. (A) P1 population rather than P2 was identified as enriched by NPCs, by flow cytometry and single cell neurosphere formation analysis was performed. (B) Quantification of single cell neurosphere formation rate in P1 and P2 populations. At least three independent experiments were quantified (***P < 0.001, *n* ≥ 3). (C) cDNA samples from two single cells were subjected to two independent library construction and sequencing in two batches. The respective Pearson correlation coefficient of two replicates was 0.97 and 0.96, indicatively minimum sequencing batch effect and reliable library construction and sequencing technology. (D) The t-SNE plot of 22 single cell transcriptome after gene filtration. Three different types of NSC/NPCs were clustered together, i.e., red (GFAP^+^/Prom1^−^/Dlx2^−^/Dcx^−^), green (Prom1^+^/GFAP^−^/Dlx2^−^/Dcx^−^), and black (Dlx2^+^/Dcx^+^/Prom1^−^/GFAP^−^). (E) Three different types of NSC/NPCs express discrete set of marker genes

To further investigate molecular characteristics of various NSC/NPCs during aging, we performed single cell RNA-seq of a discrete NSC/NPCs population from EZ and SEZ/SVZ from young and old mice. The EZ-SEZ/SVZ adjacent to striatum was micro-dissected. Single cell suspension was subjected to flow cytometry analysis. According to the size and complexity of cells, we identified two cell populations, of which one was bigger and more discrete (population 1, P1), while the other was relatively small and aggregated (population 2, P2) (Fig. 4A). In order to determine the population in which NSC/NPCs are located, single cells from EZ-SEZ/SVZ were sorted into 96-well plates as single cells by flow cytometry (Fig. 4A). We found the clonal formation rate of neurospheres from P1 was significantly higher than that of P2 (Fig. 4B). In addition, a cell surface marker CD133 (*Prom1*) was used for flow cytometry analysis of P1 and P2. Consistent with neurosphere formation potentials, P1 contains more CD133^+^ NSC/NPCs than P2 (Fig. S1). Based on the distribution pattern of P1, it is obvious that P1 still represented rather heterogeneous NSC/NPC populations (Fig. 4A). To probe the heterogeneous cellular composition of the P1 cell fraction, we randomly harvested single-cells from the P1 fraction and carried out reverse transcription using the Smart-seq2 method, followed by qPCR validation with several NSC/NPC markers, such as *Prom1*, *Gfap*, *Dlx2*, and *Dcx*. In this way, we identified 22 cells expressing these markers from both young and old brains.

Libraries of 22 single cells were constructed using Smart-seq2 method and sequenced. Sequencing samples have an average depth of 9.75 million reads, with an average transcriptome alignment rate of 49% (Table S1). We set technical replicates of different single cell cDNA libraries and sequenced different batches using the same single-cell cDNA sample, to ensure the stability of library construction and sequencing platforms. High Pearson correlation coefficient (0.96–0.97) between the replicates demonstrated our RNA-seq system was quite stable (Fig. 4C).

**Figure 5:**
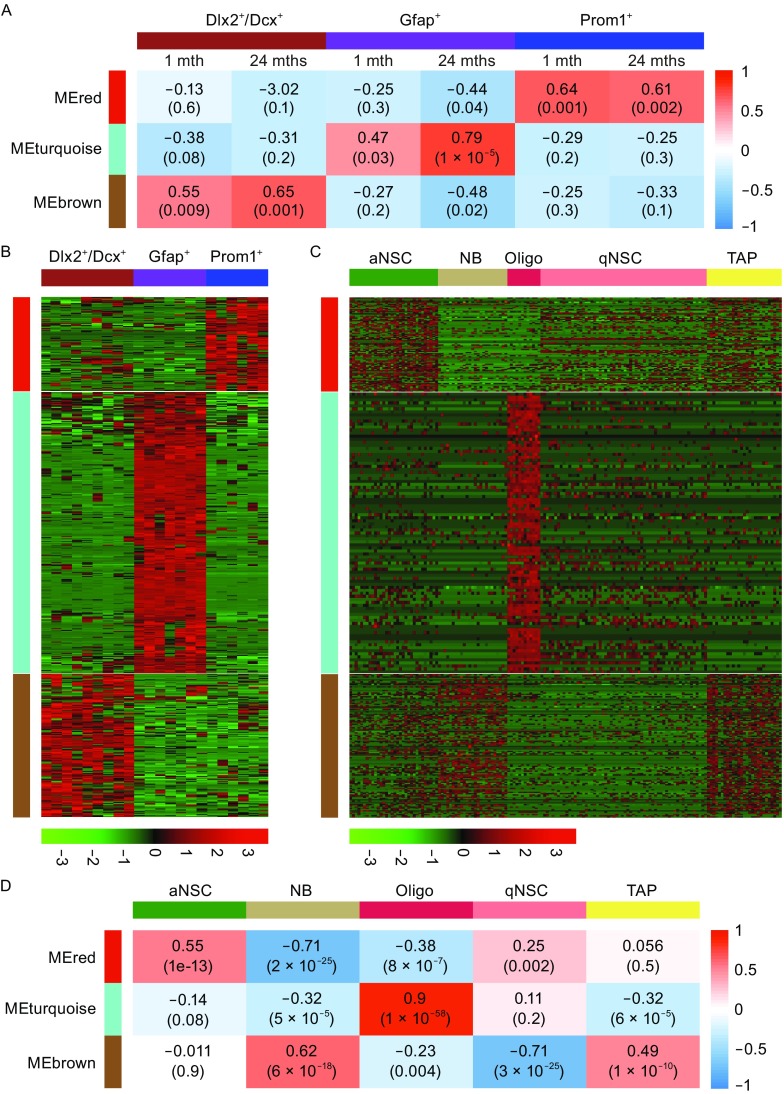
**NSC/NPC subtype identification of 22 sequenced single cells based on transcriptome analyses**. (A) WGCNA revealed three specific gene modules (red, turquoise, and brown), demonstrating enrichment in Prom1^+^, Gfap^+^, and Dlx2^+^/Dcx^+^ cells populations. (B) Heatmap of genes of red, turquoise, and brown modules in 22 sequenced single cells. (C) Cross-referencing gene expression of red, turquoise, and brown identified from our 22 single cell transcriptome, in published data set by Llorens-Bobadilla et al. in *Cell Stem Cell* (2015). (D) Correlations between the three modules with 5 published cell types by Llorens-Bobadilla et al. (2015, *Cell Stem Cell*)

We used t-distributed stochastic neighbor embedding (t-SNE) on transcriptomes of 22 cells with gene filtration. Two criteria were used, i.e., FPKM has to be bigger than 1, and the gene had to be expressed in at least 3 cells out of 22. Through this filtration 10,181 genes were obtained for t-SNE and other analyses. This unbiased analysis clearly divided 22 cells into three main clusters (Fig. 4D). The clusters were assigned to three distinct cell types based on their expression of known NSC/NPC signature genes (Fig. 4E). The Prom1^+^ cluster expressed active neural stem cell (aNSC) markers, including *Hes5*, Tlx (*Nr2e1*), *Egfr*, Glast (*Slc1a3*) and cell-cycle-related gene, *Mki67*. The Gfap^+^ cluster expressed genes, such as *Sox10*, *Olig1*, *Mag* and *Mog,* indicative of oligodendroglial lineage. The Dlx2^+^/Dcx^+^ cluster expressed *Cd24a*, *Sp9*, and *Sp8*, which is consistent of them being neuroblasts (NBs) in forebrain SEZ/SVZ. Both Prom1^+^ and NB subpopulations also expressed the well-known NSC/NPC markers, Sox2, Nestin, and Pax6. To further confirm the identity of these three cell clusters, we performed weighted gene co-expression network analyses (WGCNA) and identified three gene clusters (brown, turquoise, and red modules) that correlated well with Prom1^+^, Gfap^+^, and Dlx2^+^/Dcx^+^ cell clusters, respectively, regardless of the age of the cells (Fig. 5A and 5B). Cross reference of our data with those published (Llorens-Bobadilla et al., 2015), demonstrated that our Prom1^+^ cell-specific “red” module of genes were enriched in published aNSCs (Fig. 5C and 5D), while our Gfap+ cell-specific “turquoise” gene module was highly enriched in oligodendrocytes (Oligo), and lastly, our Dlx2^+^/Dcx^+^ cell-specific “brown” module had enriched expression in NB as well as transit amplifying progenitors (TAP) (Fig. 5C and 5D). All these cells have previously been reported to exist in the NSC/NPC pool from EZ-SEZ/SVZ in adult mouse via single cell transcriptome analyses (Luo et al., 2015; Llorens-Bobadilla et al., 2015; Dulken et al., 2017). These data suggested that although single cell transcriptome profiling is prone to carrying lots of noises, when using large gene clusters to define cell type identity, the result could be quite unambiguous and can be cross-referenced among different studies.

### Differential gene expression analysis associated with aging in different SEZ/SVZ cell subpopulations

**Figure 6:**
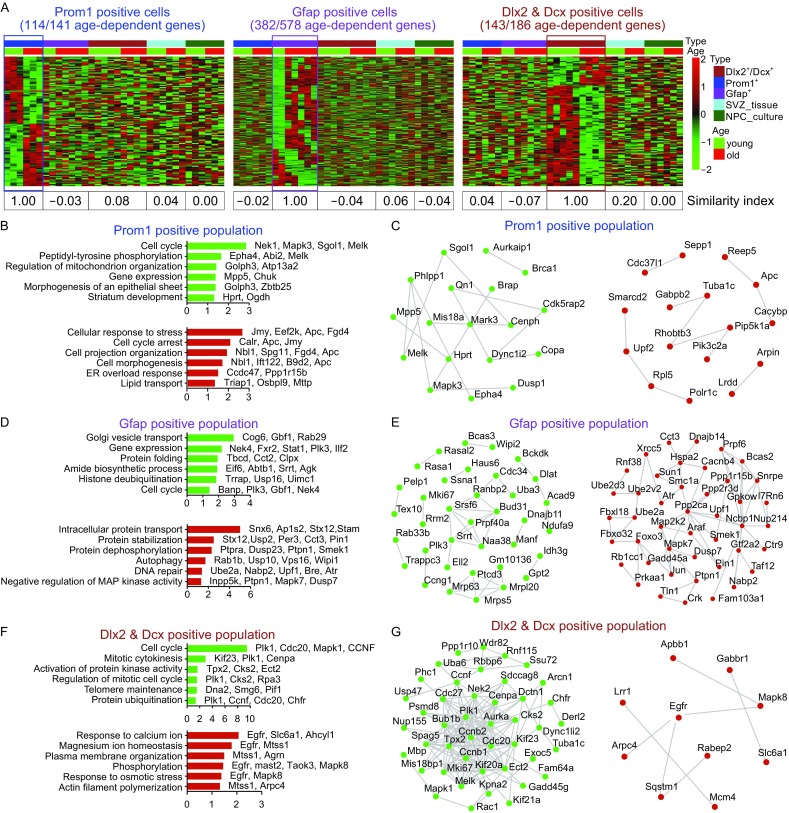
**Differential gene expression analysis revealed age-dependent molecular alterations in distinct SEZ/SVZ cell subpopulations**. (A) Heatmap of age-dependent differential genes from three distinct SEZ/SZV subpopulations, including Prom1^+^ cells, Gfap^+^ cells, and Dlx2^+^/Dcx^+^ cells respectively. Cross-data comparisons of expressions of each of these three cell type-specific age-dependent gene panels (excluding those not detected in any one of the 5 types of samples) in the rest of the 5 samples including SEZ/SVZ tissue samples and cultured NSC/NPCs. Similarity index was calculated for each of the three gene panels across 5 types of samples. (B and C) GO analyses and STRING protein-protein interaction network of age-dependent 141 genes (up- and down-related genes) in Prom1^+^ subpopulation. (D and E) GO analyses and STRING protein-protein interaction network of age-dependent 578 genes (up- and down-related genes) in Gfap^+^ subpopulation. (F and G) GO analyses and STRING protein-protein interaction network of age-dependent 186 genes (up- and down-related genes) in NBs subpopulation. Length of bars indicated the significance (−log10 transferred *P*-value, Fisher exact test)

From t-SNE analyses as well as WGCNA, it is obvious that within each of the three cell clusters (cell types), young and aged samples (cells) were segregated, which indicated distinct gene expression between old and young cells. To further understand molecular mechanisms underlying aging of each of the three clusters of cells and evaluate whether they use same or different aging regulatory gene network, we extrapolated differential gene expression in young and aged cells for each cell type, and cross referenced expression of those genes in the other cell types. We omitted genes with reads less than 5, and the gene had to be expressed in at least 3 cells out of 22. Using DEseq2 analyzing package, by comparing transcriptome of Prom1^+^/Egfr^+^ cells from 1-mth-old mouse EZ-SEZ/SVZ to that of in 24-mths-old mice (Fig. 6A), we found a total of 141 age-dependent genes. Gene ontology (GO) analyses of these genes indicated that aged cells had increased “cell cycle arrest”, increased “cellular response to stress”, increased “ER overload response”, and decreased “cell cycle” (e.g., Melk, Mapk3, etc.) as well as decreased “mitochondrial organization” genes in addition to others (Fig. 6A–C). Interestingly, these age-dependent gene clusters in Prom1^+^/Egfr^+^ cells did not show consistent age-dependency in Gfap^+^ or Dlx2^+^/Dcx^+^ cells, suggesting that they use different underlying molecular mechanisms to regulate aging (Fig. 6A). In addition, we also cross-compared transcriptomes of young and old brain tissues surrounding EZ-SEZ/SVZ as well as cultured NSC/NPCs and found that they also did not have same age-dependent gene expression as Prom1^+^ cells (Fig. 6A).

Similar analyses were carried out for Gfap^+^ cells, a total of 578 genes (408 up-regulated and 170 down-regulated) were obtained showing age-dependency (Fig. 6A, 6D, and 6E). These cells, while express Gfap mRNA, were not astrocytes, but rather oligodendrocyte lineage cells, because they expressed a large series of oligodendrocyte lineage genes but not enough astrocyte genes (Fig. S3). Aged Gfap^+^ oligo cell cluster expressed increased “autophagy”, “DNA repair” genes, while decreased expression of “cell cycle” genes, such as *Mki67* (Fig. 6A, 6D, and 6E). Aged Dlx2^+^/Dcx^+^ neuroblasts, on the other hand, increased express of “calcium, magnesium responsive genes” among others, but reduced expression of “telomere maintenance” genes as well as a lot of “cell cycle” genes including *Mki67*, *Melk*, *Mapk1* (Figs. 6A, 6F, 6G, and S2A). An NB age-depended cell cycle checkpoint gene, *Bub1*b, was chosen for immune-histochemical validation in young and aged mice, and result demonstrated that the number of Bub1b expressing cells in EZ-SEZ/SVZ (striatal side) was indeed significantly reduced in aged mice (Fig. S2B and S2C).

While age-dependent genes for each cell subtype did not share similar age-dependency in the other two cell types or brain tissues or cultured NSC/NPCs, GO analysis strongly indicated that all aged cell subtypes showed reduced “cell cycle” gene expression. This is also true for aged brain tissue samples and cultured NSC/NPCs isolated from aged brain (Figs. 6 and S4), suggesting reduced cell proliferation could be a cardinal feature for aged NSC/NPCs. Cultured NSC/NPCs increased genes in “mitochondrial electron transport” and “redox processes”, while decreased expression of genes involved in “cell cycle” and “neurogenesis” such as *Myc*, *Ascl1*, *Sox1*, *Jun*, *Tgfb2*, *Axin2*. From cross-data comparison, it is clear that aging gene network of aged brain tissue samples is relatively similar to that of Dlx2^+^/Dcx^+^ cells, and to a lesser extent, cultured NSC/NPCs (Fig. S4), suggesting that in brain tissues surrounding EZ-SEZ/SVZ, Dlx2^+^/Dcx^+^ NB might represent one of the major cell populations.

Taken together, relatively large scale, unbiased transcriptome analyses of NSC/NPC single cells, cultured cell mixture, as well as brain tissues surrounding the neurogenic zone, all demonstrated reduction of cell proliferation being associated with aged NSC/NPCs. In addition, microenvironment of aged NSC/NPCs is quite inflammatory. Given that inflammatory cytokines did not appear to slow down cell proliferation *in vitro*, it is possible that the reduction in cell cycling is an intrinsic feature of aged NSC/NPCs.

### Phosphorylation inhibition of Erk1/2 in NSCs may be involved in aging-associated decline in NSC/NPCs

**Figure 7:**
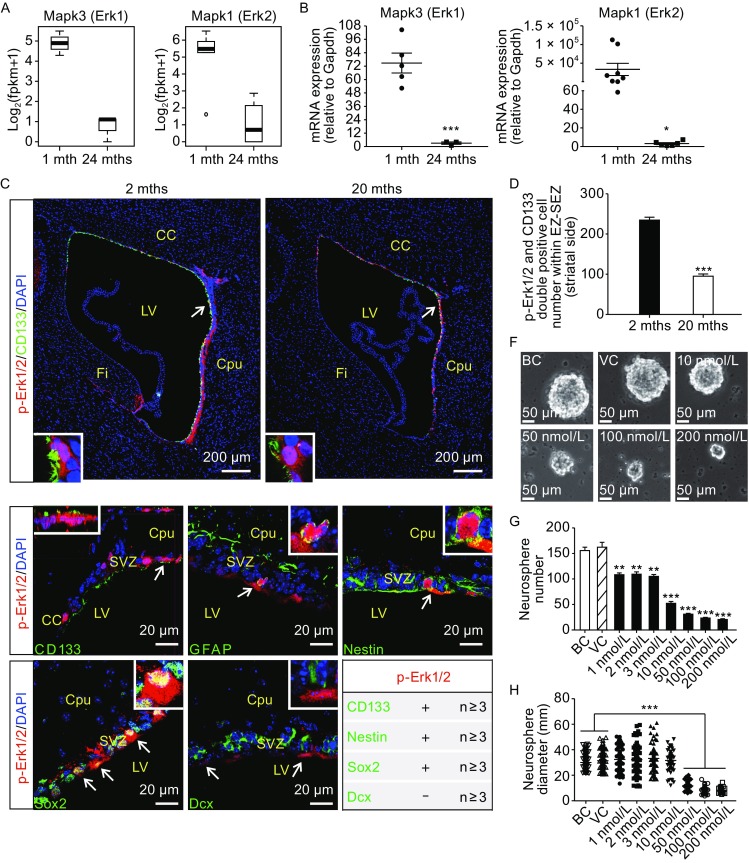
**Dampened pErk1/2-Mapkase pathway occurred in aged NSC/NPCs and suppressed clonal expansion capacity of NSC/NPCs**. (A) Box plot of Mapk3 and Mapk1, in Prom1^+^ cells and NBs, respectively, demonstrate reduction with aging based on single cell RNA-seq data. (B) qRT-PCR validated sequencing result in (A). (C) Representative micrograph of sagittal sections of mouse SEZ/SVZ from young (2 mths) and old (20 mths), immunostained with CD133 (green) and p-Erk1/2 (red). Arrows and insets indicate CD133 and p-Erk1/2 double positive cells within SEZ/SVZ. (D) Quantification of CD133^+^/p-Erk1/2^+^ cells in young (2 mths, *n* ≥ 3) and old (20 mths, *n* ≥ 3) mouse SEZ/SVZ (see MATERIALS AND METHODS). (E) Immunostaining micrographs of sagittal sections of the adult mouse SEZ/SVZ demonstrate p-Erk1/2 (red) could be co-located with the NSC markers (green, including CD133, Gfap, Nestin and Sox2), but not neuroblast marker Dcx (green). Arrows and insets indicate p-Erk1/2^+^/CD133^+^, p-Erk1/2^+^/Gfap^+^, p-Erk1/2^+^/Nestin^+^, p-Erk1/2^+^/Sox2^+^ NCS/NPCs, as well as p-Erk1/2^−^/Dcx^+^ neuroblasts within SEZ/SVZ. (F) Representative images of decreased neurosphere formation after exposure to 10 nmol/L, 50 nmol/L, 100 nmol/L, 200 nmol/L specific inhibitor of Erk1/2 phosphorylation (SCH772984), as compared to blank controls (BC) and vehicle controls (VC). (G and H) Quantification of decreased neurosphere numbers and size after exposure to SCH772984. Error bars indicate the SEM from three independent experiments. **P < 0.01; ***P < 0.001

GO analysis has revealed that Mapk cascade related genes (*Mapk3* and *Mapk1*) were down-regulated respectively in Prom1^+^ and Dlx2^+^/Dcx^+^ cells, with aging (Fig. 7A). Mapk, also known as extracellular signal-regulated kinases (ERKs), often plays a vital role in mediating intracellular signaling, including those to regulate cell proliferation. Emerging evidence suggests that Erk1/2 activity is essential to maintain proliferation of NSC/NPCs during normal neurogenesis (Satoh et al., 2011; Vithayathil et al., 2015). Down-regulation of Erk1/2 in NSC/NPCs and neuroblasts during aging may result in a decrease in overall Erk1/2 activity, leading to a decline in NSC/NPC numbers and consequently reduced production of new neurons. Quantitative RT-PCR and validated sequencing result on Erk1/2 (Fig. 7A and 7B). Moreover, quantification of cells immune-labeled with both CD133 and p-Erk1/2 (phosphorylated Erk1/2) in the EZ-SEZ/SVZ suggested that the classic MAPKase pathway was reduced in aged NSC/NPCs (Fig. 7C and 7D). Interestingly, we found pERK1/2 could be co-localized with CD133, GFAP, Sox2, Nestin single positive cells, while was never present in Dcx^+^ NBs (Fig. 7E). Given that CD24^+^ NBs cannot form clonally grown neurospheres (Coskun et al., 2008), we speculated that a lack of Erk1/2 phosphorylation might eradicate neural sphere formation capacity of NSC/NPCs. To test this, we used SCH772984 to inhibit pErk1/2 pathways and found that the capacity for NSC/NPCs to undergo clonal neurosphere forming was greatly reduced with drug treatment in a dose-dependent manner (Fig. 7F–H). Taken together, it is clear that dampened pErk1/2 pathway likely contributes to declined clonal expansion of aged NSC/NPCs.

## Discussion

Aging of the brain is a rather complex and progressive biological process. Aging of NSC/NPCs in the brain, though only represent one perspective of brain aging, is still complicated. Stem/progenitor cell aging involves not only alterations in the molecular characteristics of distinct subtypes of NSC/NPCs, but also changes in the cellular microenvironment, including blood supplies, content of cerebrospinal fluid, and interplays amongst not only NSC/NPCs but also surrounding cells. In-depth understanding of these alterations, and comprehensive analysis of linkages between them may shed light on revealing the molecular mechanisms underlying NSC/NPCs aging.

The pools of adult NSC/NPCs are extremely heterogeneous. Total RNA-seq of EZ-SEZ/SVZ tissue can only represent alterations in the overall transcription profile, while ignoring changes in individual subsets of cells. It remains uncertain that which subtypes of cells are responsible for those alterations. This is a major obstacle for studying the underlying molecular mechanisms. With single cell sequencing applied to adult neural stem cell research, some novel cell subtypes have been discovered (Luo et al., 2015). The investigation on gene signatures and alterations of distinct cell subpopulation in heterogeneous pools of adult NSCs in relation to aging may bring a new strategy for revealing novel mechanisms for stem cell aging.

As a proof of principle study, we carried out both tissue-based, cultured NSC/NPC-based, and single cell transcriptome analyses of NSC-NPCs isolated from young and aged mouse forebrain neurogenic zone. We incidentally sequenced three distinct NSC/NPC populations, but there are a lot more NSC/NPC subtypes. For example, in our published paper, we identified CD133^+^ (Prom1^+^) but Egfr negative quiescent NSCs in the ependymal region, which rely on VEGF but not bFGF as mitogens. The Prom1^+^Egfr^+^ active NSCs share some well-known NSC genes including *Nestin*, *Sox2*, *Pax6*, with neuroblasts. These features do not exist in quiescent CD133^+^ NSC populations. Moreover, our Gfap^+^ oligodendroglial lineage cells are also drastically different from the Gfap^+^ SVZ type B neural stem cells (Doetsch et al., 1999). Our study pointed out a future direction of using large scale massive parallel single cell RNA sequencing, e.g., the 10x Genomics platform (Zheng et al., 2017), to further dissect out the cellular composition and molecular characterization of the extremely heterogeneous NSC/NPC populations in the brain.

It is known that single cell RNA sequencing is prone to generating noises in the sequencing result. From this study we realized that using large gene clusters to define cell subtypes is a valid approach as compared to using a handful of biomarkers, which, based on single cell RNA sequencing, often are not that specific and still mark heterogeneous populations. It is comforting that when using large number of gene sets, it is very easy to cross-reference single cell sequencing result for cell subtype identification. Biologically, our comprehensive sequencing data sets had uniformly pointed towards one theme, i.e., aged NSC/NPCs had reduced cell proliferation. On the other hand, each of these three distinct cell populations use different molecular mechanisms to reduce cell cycling, because differential gene expression profile between young and old NSC/NPCs of a distinct subpopulation is not shared by the other subpopulations. Moreover, pErk1/2 signaling appeared to be important for the reduced capacity for clonal expansion of aged NSCs. Brain tissue-based sequencing indicated that in addition to reduction in cell cycle, aged brain expresses many immune response/inflammation related genes. It is likely that neuro-inflammation is an important element for brain aging.

Taken together, through this proof of principle study we demonstrated the power of using single cell transcriptome analyses to reveal potential molecular mechanisms underlying NSC/NPCs aging. Such fundamental studies will help us better understand brain aging and potentially reverse aging.

## Materials and methods

### Subjects

The mice used in the experiment were all C57BL/6, respectively purchased from Shanghai SLAC Laboratory Animal Co., Ltd. and Beijing Vital River Laboratory Animal Technology Co., Ltd. Mice were housed four per cage, maintained on a 12 h light/dark schedule, and allowed free access to food and water, following protocols approved by the Animal Research Committee of Tongji University School of Medicine, China.

### Neurosphere culture *in vitro*

All mice were anesthetized and euthanized in accordance with institutional guidelines. Mice brains were quickly removed from the skull and put into cold HBSS. After twice washes with cold HBSS, the brains were dissected under stereomicroscope. The lateral ventricle wall near the striatum was obtained and enzymatically digested with papain (Worthington LS003127) at 37°C water bath for 30 min. Digested cell suspension was filtered with 40 μm cell strainer. After centrifugation, the cell pellet was resuspended with DMEM-F12 (Thermo Fisher Scientific) supplemented with 1× B27 (Thermo Fisher Scientific) and plated on uncoated 96-well plate for neurosphere culture *in vitro*. Recombinant murine FGF-basic (bFGF) (10 ng/mL; Peprotech) was added every day. Five days later, the neurospheres were counted and measured.

### NPC spontaneous differentiation *in vitro*

To examine the spontaneous differentiation status of primary NPCs, we plated the single cell suspension of NPCs onto PDL (10 μg/mL in sterile distilled water; Sigma)/Laminin (5 μg/mL in DMEM-F12) coated coverslips at the density of 1 × 10^4^ cells/cm^2^. When the cell confluence reached approximately 80%, bFGF was withdrawn. After 7 days of continuous culture without bFGF, the cells were fixed with 4% PFA and performed immunostaining with GFAP and Tuj1 antibody.

### Single cell Isolation from the SVZ

As mentioned above, NPCs from the lateral ventricle wall near the striatum were obtained. After twice PBS washes, the cells were sorted using BD FACS Aria II. According to size and complexity, cells were divided into two populations, P1 and P2 population. Single cell neurosphere formation experiment showed that P1 population contained more NSCs than P2. Consequently, P1 population was sorted into dishes followed by manual picking of single cell under microscope.

### Fragment library construction and sequencing

After the generation of cDNA from a single cell through Smart-seq2 method (Picelli et al., 2014; Picelli et al., 2013), 8 ng of cDNA was used for fragment library preparation. Using the Covaris^TM^ S2 System (Covaris, Inc.), cDNA was sheared into 150 bp short fragments according to the manufacturer’s instructions. The sheared fragments were constructed into fragment library with DNA Library Prep Kit for Illumina (New England Biolabs, Inc.) according to the manufacturer’s instructions. A brief overview is as follows: first, short fragments were repaired using the end repair enzyme, then adaptors were added to the end of the fragments by T/A ligase. Afterwards, the adaptor-ligated DNA fragments were purified with AMpure XP beads and amplified for 10 cycles. Last, the libraries were used for Illumina deep sequencing. Reads were aligning to GRCm38 with HISAT2 (Kim D et al., 2015). Gene expression levels were estimated by StringTie (Pertea et al., 2015). RNA-Seq data was deposited at GSE100389.

### t-SNE

We used t-distributed stochastic neighbor embedding (t-SNE) (Laurens and Hinton, 2008) to visualize single cell RNA-Seq data. Briefly, t-SNE was calculated on all expressed genes. We used 50 principal components; perplexity = 5 and correlation as a distance measure.

### WGCNA

Genes with FPKM ≥ 1 in at least 3 samples were identified as *bona fide* expressed genes. Highly variable genes were detected by ANOVA (FDR < 0.05 for any 3 cell types and ages). 2,203 highly variable genes were supplied to weighted gene co-expression network analysis (WGCNA) as described before (Luo et al., 2015; Zhang et al., 2014). Specifically, soft-power of 7 was chosen to construct a topological overlap matrix from gene correlation network. Modules were detected by dynamic hybrid cut. Highly correlated modules (Pearson correlation of module eigengene >0.9) were merged as one module.

### Differential gene expression analysis

Differential expression between the putative groups was conducted using the R package DESeq2 (Love et al., 2014), genes which were expressed at least 5 read counts in 3 samples would take into consideration. To identify significant genes, we select genes with criteria of *P*-values < 0.05 and absolute values of the logarithm (to basis 2) of the fold change (LogFC) > 1. Functional enrichment analysis based on gene ontology (GO) database was performed by using David 6.7 program (http://david.abcc.ncifcrf.gov/) and protein-protein interactions (PPI) network of DEGs were constructed using STRING database (http://www.string-db.org/). Similarity index was calculated by Pearson correlation coefficient. Briefly, we transform subtype-specific differential expressed genes into 3 levels, 1 and −1 for significantly up- or down-regulated genes respectively and 0 for insignificant genes. Pearson correlation coefficient was calculated using transformed gene alteration degree.

### Immunostaining

The tissue slices or cultured samples were treated with methanol or 4% PFA for 10 min at −20°C. After three washes, all slides were incubated in blocking buffer (10% BSA and 5% normal donkey serum and 0.2% triton X-100 in PBS) for 1 h at room temperature. Slides or coverslips in 24-well plate were immersed in primary antibody buffer (200 µL per slide or 300 µL per well) for incubation overnight at 4°C. The primary antibodies that were used in this study are: anti-GFAP (Dako) 1:1000, anti-Tuj1 (R&D) 1:500, anti-doublecortin (Cell Signaling Technology) 1:400 (Santa Cruz) 1:1000, anti-Ki67 (Abcam) 1:200, anti-sox2 (Abcam) 1:200, anti-Bub1b (Abcam) 1:100, anti-p-Erk1/2 (Cell Signaling Technology) 1:200, anti-CD133 (Ebioscience) 1:500, anti-Nestin (Millipore) 1:1000, anti-Iba1 (Abcam) 1:200. The following day, the coverslips and slides were washed three times. Then they were incubated in appropriate Alexa 488-, Alexa 568- or Cy3-conjugated secondary antibodies (Thermo Fisher Scientific) for 1 h at room temperature at 1:1000 dilution. DAPI staining was used to label the nuclei. After mounting overnight at room temperature, the slides were examined via Nikon fluorescent microscopes or a confocal system (Leica; TCS SP5 II). Quantification of Tuj1^+^, GFAP^+^, Ki67^+^, Sox2^+^, Bub1b^+^, Bub1b^+^/Dcx^+^, Iba1^+^ and CD133^+^/p-Erk1/2^+^ cells were performed by counting the labeled cells within the SVZ from three independent experiments. Statistical analysis was performed using unpaired *t* test.

### Quantification of immunohistochemical staining

Each experimental group contained at least 3 mice, 12 serial sections (sagittal section, 10 μm) were chosen for subsequent immunostaining per mouse, according to similar anatomical locations among each mouse.

### Quantitative real-time PCR

Quantitative real-time PCR (qRT-PCR) was performed using TaqMan real-time PCR system (Thermo Fisher Scientific) and SYBGreen real-time PCR system (TaKaRa). 1 μL of cDNAs were used as template to run 20 μL real-time PCR reactions to check the expression of house-keeping gene GAPDH and other genes to be detected, including *CD133*, *GFAP*, *Dlx2*, *Dcx*, *Ncam1*, *Mapk1*, and *Mapk3*. All reactions were triplicated. The PCR was done as follows in an AB7500 thermocycler (Applied Biosystems) with 96-well plates: 95°C for 10 min; then 40 cycles of 95°C for 15 s; and 60°C; for 1 min. The primers of quantitative real-time PCR are shown in Table S2.

### The inhibitor of Erk1/2 administration neurosphere formation *in vitro*

The single cell suspension derived from two 2-mths-old C57BL/6 was plated on 9 wells of two 6-well plate. The culture medium is DMEM-F12 (Thermo Fisher Scientific) supplemented with 1× B27 (Thermo Fisher Scientific). And bFGF (10 ng/mL; Peprotech) was daily added. Erk1/2 inhibitors were respectively added into 7 wells at the final concentration of 1 nmol/L, 2 nmol/L, 3 nmol/L, 10 nmol/L, 50 nmol/L, 100 nmol/L, and 200 nmol/L. Vehicle control was added the same amount of inhibitor solvent while blank control was added nothing but culture medium. Five days later, the neurospheres were counted and measured. The experiments were triplicated.

### Graphics

Unless otherwise specified, plots were generated in R or GraphPad Prism 5.

## Electronic supplementary material

Below is the link to the electronic supplementary material.

Supplementary material 1 (PDF 780 kb)

Supplementary material 2 (XLSX 9 kb)

Supplementary material 3 (XLSX 9 kb)
