# Supplementary material for: Single-cell transcriptomics reveals gene signatures and alterations associated with aging in distinct neural stem/progenitor cell subpopulations
Source: Protein Cell. 2017 Jul 26;9(4):351–64. doi: 10.1007/s13238-017-0450-2 (PMC5876182; doi:10.1007/s13238-017-0450-2)
Supplement: Supplementary file 1 — Supplementary material 1 (PDF 780 kb) [file 13238_2017_450_MOESM1_ESM.pdf]

Supplementary materials

Table legends

Table S1. Total reads, mapped reads, and mapping rates for all cells (related to Figure 4)

Table S2. Primers used for qPCR in this study

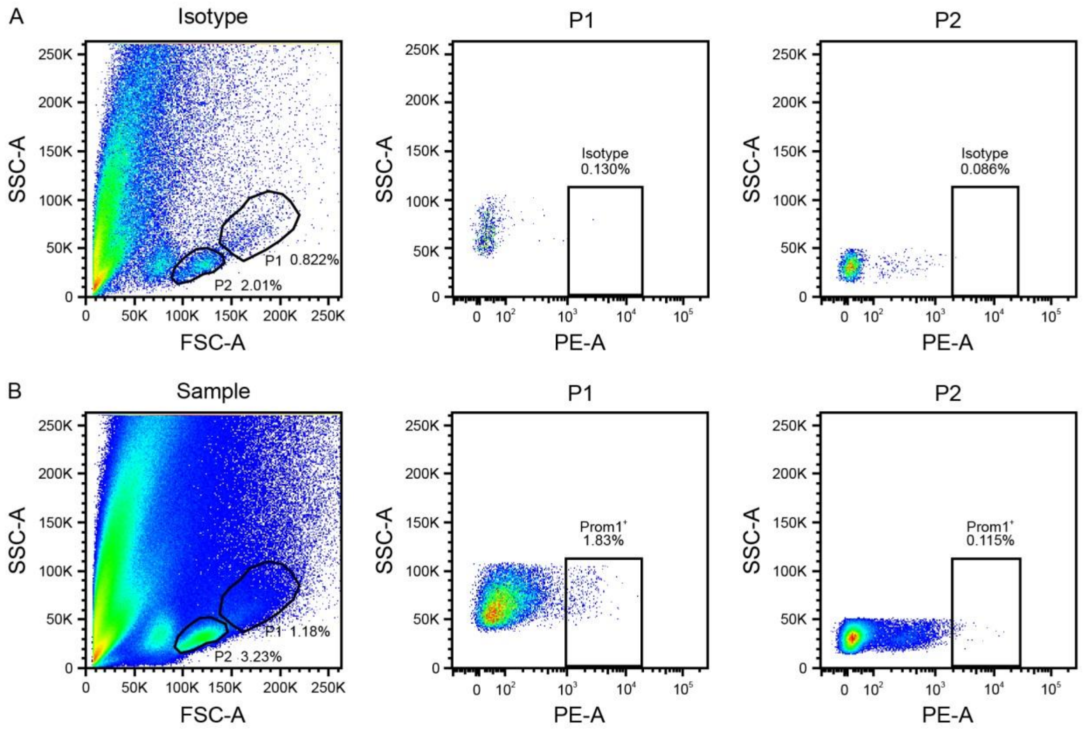

Sup. F1 Shi *et al.*

Supplement figure 1. NSC/NPC P1 population contained more CD133+ cells than P2 population

(A) Flow cytometry analysis of isotype samples to determine gating of the samples. (B) Flow cytometry analysis of samples indicated that P1 population contained more CD133+ cells than P2.

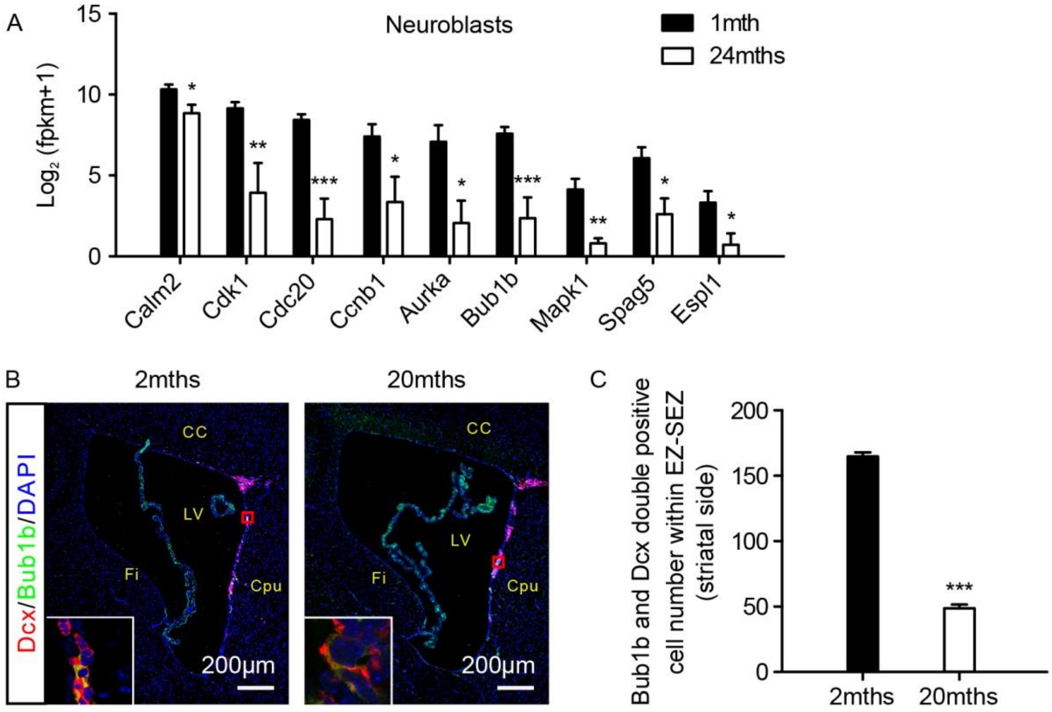

Sup. F2 Shi *et al.*

Supplement figure 2. Validation of age-dependent gene expression in Dlx2+/Dcx+ neuroblasts

(A) Expression of 9 age-dependent cell cycle genes in Dlx2+/Dcx+ cells based on single cell RNA-seq data. (B) A representative sagittal section from young (2mths) and old (20mths) mouse SEZ/SVZ stained with Bub1b (green) and Dcx (red). The insets indicate Dcx and Bub1b double positive cells. (C) Quantification of Bub1b and Dcx double positive cells in young (2mths, n⩾3) and old (20mths, n⩾3) mouse SEZ/SVZ. Error bars indicate the SEM from at least three independent experiments. ***, P <0.001. CC, corpus callosum; Cpu, caudate putamen; LV, lateral ventricle; Fi, fimbria of the hippocampus.

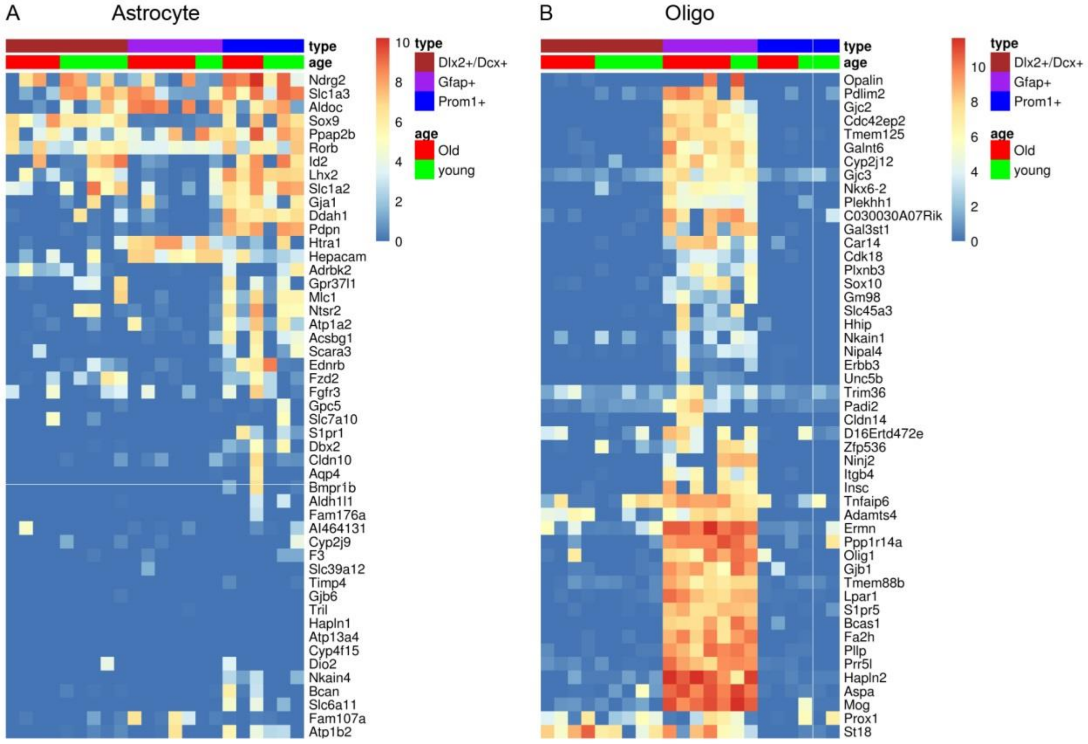

Sup. F3 Shi *et al.*

Supplement figure 3. GFAP-positive cells have oligodendrocyte cell identity rather than astrocytes

Gene expression heatmaps of reported astrocyte-specific (A) and oligodendrocyte-specific (B) genes in our 22 sequenced cells, which belong to three types of cells. Result showed that our Gfap+ cells expressed a lot more oligodendrocyte-lineage genes than astrocyte-specific genes, therefore it should be defined as oligodendroglial lineage cells.

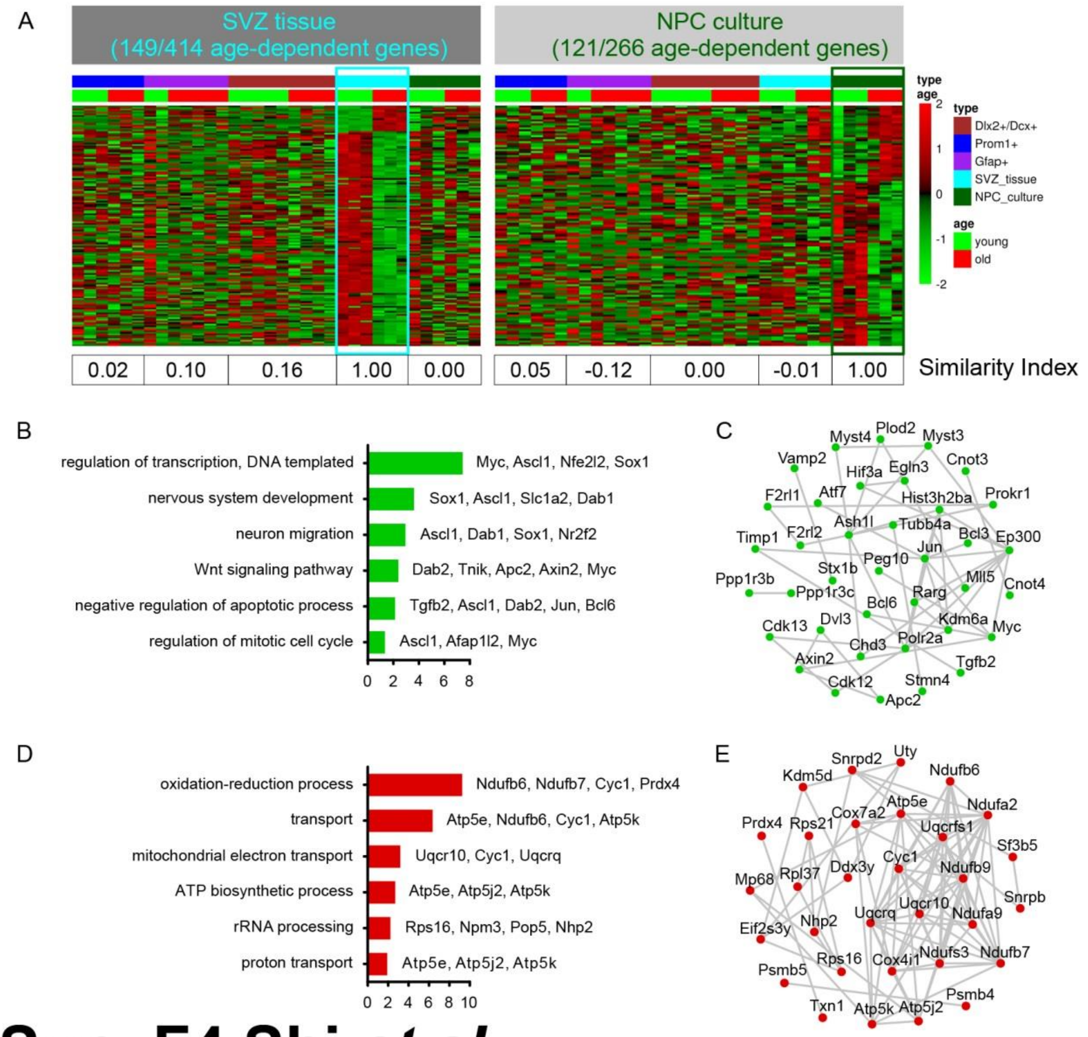

Sup. F4 Shi *et al.*

Supplement figure 4. GO analysis revealed age-dependent gene alterations of cultured NSC/NPCs in vitro

(A) Heatmap of differential genes from EZ-SEZ/SVZ tissue and cultured NSC/NPCs mapping to 5 different sample types. Similarity index suggested that there was some degrees of similarity in age-dependent genes in Dlx2+Dcx+ neuroblasts and EZ-SEZ/SVZ tissue samples. GO analyses and STRING protein-protein interaction network of down-regulated age-dependent genes (B-C) and up-regulated age-dependent genes (D-E) from cultured NSC/NPCs. Length of bars indicated the significance (−log10 transferred P-value, Fisher exact test).
